# Impacts of environment and forest type on the relationship between stand structure diversity and productivity in natural mixed forests

**DOI:** 10.1371/journal.pone.0329730

**Published:** 2025-08-14

**Authors:** Jianwu Wang, Sen Xu, Binglou Xie, Chenghao Zhu, Xiaonan Wu, Kang Ji, Qun Du

**Affiliations:** 1 Zhejiang Forest Resources Monitoring Center, Hangzhou, China; 2 Zhejiang Forestry Survey, Planning and Design Co., Ltd., Hangzhou, China; Chinese Academy of Sciences, CHINA

## Abstract

Forest productivity reflects forest growth quality and forms the basis for achieving forest service functions. The relationships between forest productivity and stand structure has been extensively studied, but it is still unclear whether environmental factors affect the relationship and how their relationships vary under the influence of stand type and environmental factors. A fixed monitoring dataset from 972 plots of natural mixed forests (including coniferous, broad-leaved, and coniferous and broad-leaved mixed forests) in Zhejiang Province was used. We analyzed the relationship between stand structure diversity (composition diversity and size differentiation diversity) and productivity in the different forest types and their influencing factors. Species richness, coefficient of the diameter at breast height (DBH) variation, and the DBH Shannonâ€’Wiener index were significantly positively correlated with productivity (P < 0.01). Environmental factors such as terrain and meteorology were associated with stand productivity and structure diversity. Considering the effects of environmental factors and stand density, it was evident that stand density was the primary direct factor influencing coniferous forest productivity ($r_{\partial }$ = 0.635), and terrain exerted a substantial indirect effect on productivity through stand density ($r_{\partial }$ = 0.202). In broad-leaved forests, topography ($r_{\partial }$ = −0.161), size differentiation diversity ($r_{\partial }$ = −0.519), and stand density ($r_{\partial }$ = 0.954) were the primary factors influencing productivity, with climate exerting a significant indirect effect via stand density and size differentiation diversity. In coniferous and broad-leaved mixed forests, stand density ($r_{\partial }$ = 0.862), size differentiation diversity ($r_{\partial }$ = −0.424), and composition diversity ($r_{\partial }$ = 0.260) were all significantly correlated with productivity. The effect of structure diversity on productivity in natural broad-leaved mixed forests in Zhejiang Province was modulated by environmental factors and stand density. Our research deepens understandings of the factors driving productivity in natural broad-leaved mixed forests and offers a theoretical foundation for sustainable development.

## Introduction

Stand structure diversity is a fundamental metric for assessing the relative richness and uniformity of structural attributes, reflecting the diversity and complexity of stands in both horizontal and vertical directions [1,2]. Stand structural diversity can be characterized and described horizontally through compositional diversity and size differentiation diversity. Compositional diversity refers to the extent of species diversity and distribution of relative abundance among species [3]. Size differentiation diversity characterizes the degree of variation in tree dimensions based on girth and height [4,5]. Diversity indices using diameter at breast height (DBH) as a comparative factor are common, such as the coefficient of DBH variation, DBH Shannonâ€’Wiener index, neighborhood comparison, etc. [6,7].

In ongoing discussions regarding the relationship between stand structure diversity and productivity, the majority of studies suggest that mixed forests exhibit higher productivity levels than single-species forests [8–11]. There is a positive correlation between stand composition diversity and forest productivity [12–15]. However, some studies have identified a positive correlation between diversity and productivity within a specific range; beyond this threshold, the contribution of diversity to productivity gradually diminishes [16]. As mentioned above, the relationship between stand structural diversity and productivity varies among studies, it is urgent to further strengthen the research on the relationship between diversity and productivity. Complex structures can lead to differences in resource heterogeneity or resource utilization efficiency, which may be the cause of this phenomenon [17]. During forest succession, forests can improve productivity by acquiring environmental resources and accumulating biomass [18,19]. Terrain factors, including slope, altitude, and aspect, indirectly influence the species composition and physiological constraints of forest stands by redistributing light, water, and thermal resources. Moreover, meteorological factors are directly related to the efficiency of forest resource acquisition and utilization [20–23]. Ni *et al*. [24] quantified the impact of stand characteristics and environmental factors on the productivity of Masson pine plantations and believed that temperature and precipitation had a positive impact on productivity. However, Wu *et al*. [25] contend that climate (temperature and precipitation) indirectly influences forest productivity by altering the size and structure of forest stands. Species interact with the environment and gradually form a relatively stable community structure. In a study of the structure of karst forests in southwest China, it was found that altitude was significantly negatively correlated with species diversity and species structure complexity, but slope was significantly negatively correlated with species diversity [21]. However, the study by Zhang *et al*. [26] showed that altitude has the smallest contribution to forest biomass in Guizhou Province among all topographic factors. It can be seen that environmental factors play a key role in driving community structure and plant growth. However, the constraints of complex environmental factors on the relationship between stand structure diversity and productivity are still unclear, and this interaction may change the impact of a single variable [24,27]. Therefore, further elucidation of the association between diversity and productivity within forest ecosystems and their interplay with environmental factors is necessary.

Natural forests are stands formed by natural regeneration under natural environmental conditions. After long-term natural succession, they form a relatively stable and coordinated ecosystem that plays an important role in maintaining biodiversity, regulating climate, and sequestering carbon and oxygen [28,29]. In order to better protect natural forests, upon implementing the “Natural Forest Resources Conservation Project," China introduced a series of relevant policies that further promoted the protection and restoration of domestic natural forests. Although both the overall forest area and quality have significantly improved, due to insufficient enforcement of natural forest protection policies and outdated technical measures, natural forests still face challenges such as low quality and slow ecological recovery [30,31]. Clarifying the driving mechanisms of natural forest productivity and understanding the complex coupling relationships between forest structural diversity and environmental factors in terms of productivity are of great significance for developing effective natural forest management strategies and ensuring the healthy development of natural forest ecosystems.

This study was based on continuous inventory data of forest resources in Zhejiang Province from 2014 to 2019. A total of 972 natural mixed forest plots were used as the research objects to analyze the relationship between forest structure diversity and productivity of different forest stand types (coniferous, broad-leaved, and coniferous and broad-leaved mixed forests). Our main objectives were to explore the following two issues: (1) Does stand structure diversity (composition diversity and size differentiation diversity) have a significant effect on forest productivity? (2) Do terrain and climate affect stand structure diversity and productivity? If there is an effect, how do terrain and climate affect stand structure diversity and productivity?Based on the aforementioned issues, we propose the following hypotheses: (1) Size differentiation diversity suppresses forest productivity by intensifying resource com-petition, whereas composition diversity positively influences productivity only in mixed coniferous and broad-leaved forests. (2) Topographic factors directly or indirectly constrain productivity via environmental filtering, with forest type differentiation observed in their pathways of influence on productivity. (3) Climate stress reduces productivity through the sequential mediation of ’density-structural diversity’. (4) For-est stand density serves as the core regulator, directly affecting resource availability and exerting a positive influence on productivity, while also mediating the effects of environmental factors.

To address these questions and verify our hypothesis, we focused on three natural mixed forest types: coniferous forests, broad-leaved forests, and coniferous broad-leaved mixed forests. These mixed forests are widely distributed in the region, and multiple continuous fixed monitoring plots were available for each forest type. We used general linear models and Pearson correlations to analyze the individual effects of stand structural diversity and environmental factors on productivity. We hypothesized that topography and climate could mediate the relationship between structural diversity and productivity, and constructed a path model to clarify the direct and indirect effects of topography, climate, stand structural diversity, and stand density on the productivity of the three types of forests.

## Materials and methods

### Study area

    Zhejiang Province (27^°^06’N—31^°^11’N, 118^°^01’E—123^°^10’E) is situated in the central part of the subtropical zone, characterized by a subtropical monsoon climate. The topography gradually descends from southwestâ€’northeast, with mountainous terrain dominating the southwest at an average altitude > 1000 m. In the central region, the hills < 500 m alternate between basins of various sizes. The northeast comprises an alluvial plain < 10 m elevation and features a dense river network. The predominant soil types include zonal soils such as red and yellow soils, along with minor occurrences of purple and lime soils. Zhejiang Province has abundant forest resources within its boundaries and falls under the subtropical evergreen broad-leaved forest zone classification. According to the Zhejiang Forest Resources Report (2019), the total forest area in Zhejiang Province spans approximately 6.0788 million ha, corresponding to a forest coverage rate of 61.15%. Among these, natural forests encompass approximately 3.6916 million ha, accounting for approximately 60.73% of the forested area.

### Research materials

The design-based sampling method is used for the fixed sampling plots of the continuous forest resource inventory in Zhejiang Province. The sampling plots are arranged at a spacing of 4km×6km. The total number of sampling plots is 4252. The sampling plots are 800 m^2^ squares. Fixed stakes are buried at the top corners of each plot to f facilitate later relocation. Each tree and shrub species (defined as shrubs with a distinct trunk, certain branch height, and a tree height reaching ≥ 5 m) exhibiting a DBH ≥ 5 cm and normal growth within the sample plot were identified using unique aluminum plaques. Starting in May each year (the initial year is 2004), a survey was conducted on the sample plot to record basic environmental characteristics such as terrain, soil factors, and understory vegetation factors. Simultaneously, each sample tree with aluminum plaques was measured to record the DBH and spatial position coordinates. The DBH and spatial position coordinates were recorded for recruitment trees with a 5 cm DBH.

This study was based on continuous forest resource inventory data from two periods in 2014 and 2019, including sample tree data (tree species, DBH, spatial position, etc.) and terrain data (altitude, aspect, and slope). A total of 972 plots with natural mixed forests in both phases (including only young, middle-aged, and near- mature forests, mature forests and over-mature forests are excluded because of their small number.) were selected based on plot type, vegetation type, age group, and origin. No human factors caused damage to the sample plots, and there were no serious natural disasters. Raster data for the climate variables (mean annual temperature [MAT], mean annual precipitation [MAP], number of frost-free days [NFFD], and Hargreaves climatic moisture deficit [CMD]) were obtained from WorldClim (https://www.worldclim.org accessed on February 16, 2024) [32]. The meteorological conditions of the sample plots were extracted using the “Point Multi Value Extraction" tool in ArcGIS 10.8 software.

### Statistical analysis

    The volume of all living trees with a DBH ≥ 5 cm in each plot was calculated individually and the total volume of living trees with a DBH ≥ 5 cm in the plot was calculated.A one-way volume model was used to calculate the individual tree stem volumes and then summed to obtain the stand volume.


$$V=a(a_{1}+b_{1}DBH{)}^{b}(a_{2}\frac{b_{2}}{a_{1}+b_{1}DBH+k})$$

where $a_{1},b_{1},a_{2},b_{2}$*, k, a, b,* and *c* are parameters (Table 1). In constructing the one-way volume model, Zhejiang Province was divided into four regions: northwestern, central, eastern, and southern, ensuring that the values of $a_{2},b_{2}$, and *k* were matched according to the region where the plots were located.

**Table 1:** Parameters of one-variable tree volume model.

Regions	$a_{1}$	$b_{1}$	$a_{2}$	$b_{2}$	*k*	*a*	*b*	*c*
Northwest	−0.1334	0.9800	37.3295	−2053.1848	60	$5.0479\cdot 10^{-5}$	1.9085	0.9908
Middle			62.0125	−8025.0582	138			
East			37.9044	−2183.3093	84			
South			58.2082	−6994.7390	128			

Stand productivity was assessed based on the annual volume growth per unit area, and forest stand density was expressed as the basal area density per unit area. Composition diversity indices encompassed richness, Pielou evenness index, inverse Simpson diversity index, and mingling [33–35]. Size differentiation diversity indices included the coefficient of DBH variation, DBH Shannonâ€’Wiener index, and neighborhood comparison [36–38]. The calculation for the stand neighborhood comparison relied on that for the individual tree neighborhood comparison within the sample plot. Detailed methods for calculating the composition and size differentiation diversity are presented in Table 2. All indicators are based on the data from the year 2014.

**Table 2:** Calculation formulas for composition diversity and size different diversity.

Index	Formula	Definition
Richness	S=Ns	*Ns* is the total number of species in the sample plot.
Pielou evenness index	$J=\frac{-\sum_{i=1}^{Ns}P_{i}^{2}\times \ln P_{i}}{\ln Ns}$	*Ns* is the total number of species in the sample plot, *P*_*i*_ is the proportion of individuals in the *i* species to the total number of individuals across all species.
Inverse Simpison diversity index	$ID=\frac{1}{1-\sum_{i=1}^{Ns}P_{i}^{2}}$	*Ns* is the total number of species in the sample plot, *P*_*i*_ is the proportion of individuals in the *i* species to the total number of individuals across all species.
Mingling	$M=\frac{1}{N}\sum \frac{1}{n}\sum_{j=1}^{n}v_{ij}$	*n* is the number of adjacent trees (In this study, n=4) ; *$v_{ij}$* is a discrete variable, when the *i*-th target tree and its adjacent tree *j* are of the same species, the value of $v_{ij}$ is 1, otherwise it is 0.
Coefficient of DBH variation	$DBH_{cv}=\frac{\sqrt{\frac{1}{N-1}(DBH_{k}-\mu {)}^{2}}}{\mu }$eak ×100%	*DBH*_*k*_ is the chest diameter value of the *k*-th individual in the plot , *μ* is the average DBH of all individuals in the sample plot, *N* is the total number of individuals in the sample plot.
DBH Shannon-Wiener index	$DBH_{cv}=-\sum_{i=1}^{Nd}\frac{n_{i}}{N}\times \ln\frac{n_{i}}{N}$	*Nd* is the total number of DBH grades within the sample plot (dividing tree DBH into grades at 1cm intervals), *n*_*i*_ is the number of individuals of the *i*-th species in the sample plot, *N* is the total number of individuals in the sample plot.
Neighborhood comparison	$U=-\frac{1}{N}\sum \frac{1}{n}\sum_{j=1}^{n}\frac{n_{i}}{N}k_{ij}$	*n* is the number of adjacent trees (In this study, n=4); *k*_*ij*_ is a discrete variable, when the *i*-th target tree is smaller than the adjacent tree *j*, the value of *k*_*ij*_ is 0, otherwise it is 1.

Density, productivity, and structure diversity characteristics of different forest types (coniferous, broad-leaved, and coniferous and broad-leaved mixed forests) were analyzed using SPSS 23.0. The coefficient of variation (CV) for each indicator was also calculated. To analyze the variation amplitudes of each index across all plots in 2014, we also cal-culated the CV for each index. CV ≤ 20.0% indicated weak variability, 20.0% < CV < 50.0% indicated moderate variability, and CV ≥ 50.0% indicated strong variability [39]. General linear models were employed to explore the relationship between structure diversity and productivity among different forest stand types. Correlation analysis and examination of the relationships between environmental factors and forest structure diversity, density, and productivity were conducted using TBtools [40]. The partial least squares path model (PLS-PM) was used to elucidate the direct and indirect effects of terrain, climate, stand structure diversity, and stand density on forest productivity. A collinearity test was conducted on the manifest variables and indicators with variance inflation factor > 10 were subsequently removed. Subsequently, the data were divided into four latent variables: terrain (altitude, aspect, and slope), meteorology (MAT, MAP, NFFD, and CMD), compositional diversity (richness, Pielou evenness index, inverse Simpson diversity, and mingling), and size differentiation diversity (coefficient of DBH variation, DBH Shannonâ€’Wiener index, and neighborhood comparison). Iterative algorithms were used to estimate the load values of latent and indicator variables for each latent variable. For indicators with negative load values, the opposite direction was used to ensure that the load values in the model were positive [41]. The PLS-PM was built using Smart PLS 4.

Based on the PLS-PM analysis results, to test the possible nonlinear relationship (e.g., unimodal patterns) between density and productivity, we performed a quadratic polynomial regression analysis. Additionally, we applied LOESS non-parametric smoothing (locally weighted regression, span = 0.8) to visualize the density-productivity relationship without assuming a predefined functional form. The significance of the quadratic model was assessed by examining the p-value of the quadratic term (p <  0.05 was considered significant). Furthermore, we compared the goodness of fit (*R*^2^) of the linear model (including only the density term) and the quadratic model to evaluate the improvement in explanatory power contributed by the nonlinear components. Finally, we calculated the theoretical peak density based on the coefficients of the quadratic model and verified whether this peak fell within the observed density range.

## Results

### Productivity and density characteristics of natural broad-leaved forest stands

The CV for stand density in all three forest stand types exceeded 50%, indicating significant variability, with the highest CV in coniferous and broad-leaved mixed forests and the lowest in coniferous forests (Table 3). The CV for forest productivity ranged from 35.35â€’42.15%, representing moderate variability, with the highest CV in coniferous and broad-leaved mixed forests and the lowest in broad-leaved forests. Productivity of coniferous and broad-leaved mixed forests was 1.06-times higher than that of broad-leaved forests.

**Table 3:** Productivity and density of natural broad-leaved forest stands.

Forest stand types	Indicators	Range	Mean	Standard deviation	Coefficient of variation %
Coniferous forest	Stand density ${\text{cm}}^{2}\cdot {\text{m}}^{-2}$	1.00∼44.03	13.06	8.14	62.34
	Productivity ${\text{m}}^{3}\cdot {\text{hm}}^{2}$	1.21∼8.89	4.50	1.90	42.15
Broad-leaved forest	Stand density ${\text{cm}}^{2}\cdot {\text{m}}^{-2}$	0.26∼58.81	14.84	8.71	58.69
	Productivity ${\text{m}}^{3}\cdot {\text{hm}}^{2}$	1.57∼8.25	4.74	1.67	35.35
Coniferous and broad-leaved mixed forest	Stand density ${\text{cm}}^{2}\cdot {\text{m}}^{-2}$	2.55∼58.34	15.22	10.57	69.49
	Productivity ${\text{m}}^{3}\cdot {\text{hm}}^{2}$	1.60∼9.01	5.01	1.81	36.19

### Diverse characteristics of natural broad-leaved forest in terms of stand structure diversity

#### Composition diversity.

The compositional diversity of forest stands in broad-leaved and coniferous and broad-leaved mixed forests was significantly higher than that of coniferous forests, with broad-leaved forests exhibiting the highest diversity (Table 4). Based on analysis of the CV, the CV values for richness, inverse Simpson diversity index, and mingling in broad-leaved forests and coniferous and broad-leaved mixed forests ranged from 22.82â€’46.73%, indicating moderately variable indices; whereas, the CV values for the Pielou evenness index were 16.26% and 12.82%, respectively, reflecting weakly variable indices.

**Table 4:** Composition diversity of natural broad-leaved forest stands.

Forest stand types	Indicators	Range	Mean	Standard deviation	Coefficient of variation %
Coniferous forest	Richness	2∼24	9.31	4.30	46.19
	Pielou evenness index	0.05∼0.99	0.58	0.18	31.54
	Inverse Simpison	1.01∼8.69	2.98	1.68	56.35
	Mingling	0.02∼0.79	0.44	0.18	40.81
Broad-leaved forest	Richness	3∼29	15.65	4.69	29.93
	Pielou evenness index	0.20∼0.94	0.72	0.12	16.26
	Inverse Simpison	1.18∼17.29	5.53	2.50	45.14
	Mingling	0.15∼0.91	0.61	0.14	23.25
Coniferous and broad-leaved mixed forest	Richness	3∼24	14.02	5.30	37.80
	Pielou evenness index	0.34∼0.91	0.73	0.09	12.82
	Inverse Simpison	1.40∼14.00	5.25	2.45	46.73
	Mingling	0.21∼0.87	0.60	0.14	22.82

#### Size differentiation diversity.

Among the three forest stand types, the CV of the coefficient of DBH variation in coniferous and broad-leaved mixed forests was higher than that in broad-leaved and coniferous forests (Table 5). Additionally, the DBH Shannonâ€’Wiener index was highest in coniferous forests. No significant differences were observed among the three forest stand types when their neighborhoods were compared. Based on the CV analysis, except for the coefficient of DBH variation, which served as a moderate indicator of variation, all indices displayed a CV < 20%, indicating weak variability.

**Table 5:** Size different diversity of natural broad-leaved forest stands.

Forest stand types	Indicators	Range	Mean	Standard deviation	Coefficient of variation %
Coniferous forest	Coeffcient of DBH variation	20.93∼72.38	39.31	9.02	22.61
	DBH Shannon-Wiener index	1.49∼3.01	2.39	0.30	12.40
	Neighborhood comparison	0.04∼0.56	0.44	0.08	17.65
Broad-leaved forest	Coeffcient of DBH variation	9.99∼99.90	40.15	12.37	30.80
	DBH Shannon-Wiener index	0.65∼3.29	2.17	0.41	19.06
	Neighborhood comparison	0.08∼0.55	0.44	0.07	16.30
Coniferous and broad-leaved mixed forest	Coeffcient of DBH variation	15.87∼71.20	42.56	10.43	24.50
	DBH Shannon-Wiener index	1.30∼3.06	2.26	0.31	13.90
	Neighborhood comparison	0.27∼0.53	0.45	0.05	12.20

#### Relationship between structure diversity of natural broad-leaved forest stands and productivity.

**Fig 1:**
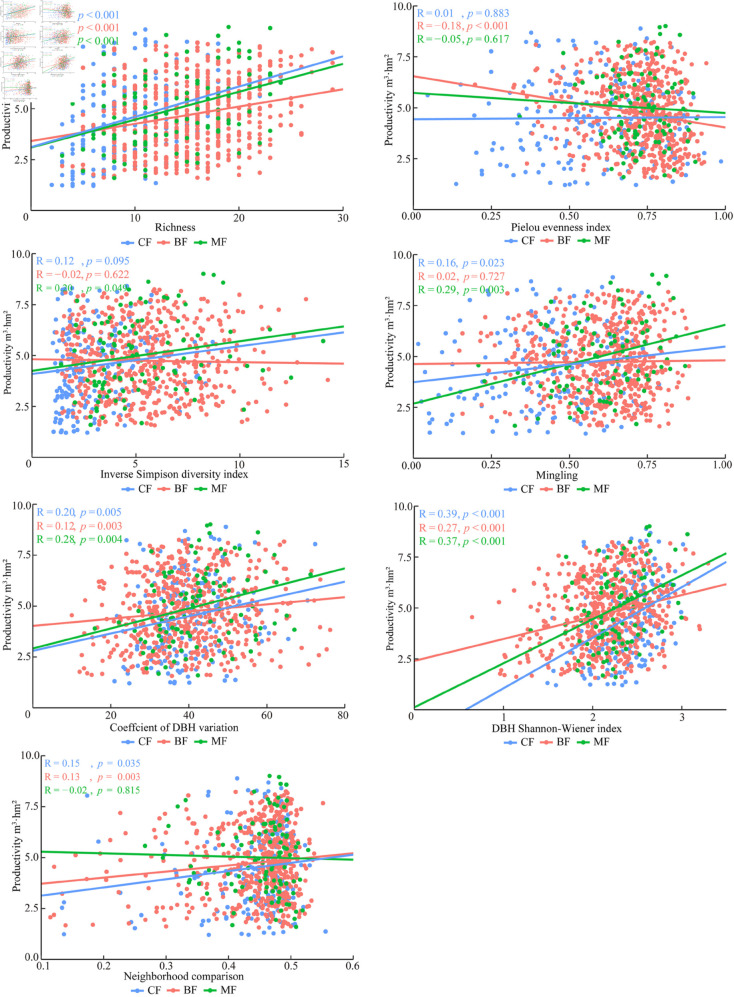
Linear regression analysis of the relationship between structural diversity and productivity in natural broad-leaved mixed forests. CF: Coniferous forest; BF: Broad-leaved forest; MF: Coniferous and broad-leaved mixed forest.

Stand structure diversity exhibited an overall positive correlation with productivity (Fig 1). A highly significant correlation (P < 0.01) was observed between richness, coefficient of DBH variation, DBH Shannonâ€’Wiener index, and productivity across the three forest stand types. In coniferous and coniferous and broad-leaved mixed forests, the Shannonâ€’Wiener index and mingling were significantly positively correlated with productivity (P < 0.05); whereas, a highly significant correlation was found between mingling and productivity in coniferous and broad-leaved mixed forests (P < 0.01). The Pielou evenness index demonstrated a significant positive correlation with productivity only in broad-leaved forests (P <  0.01); whereas, the inverse Simpson diversity index displayed a significant negative correlation with productivity only in coniferous and broad-leaved mixed forests (P < 0.05). There was a significant positive correlation (P < 0.05) between the neighborhood comparison of coniferous and broad-leaved forests, as well as their respective productivities, along with a highly significant correlation (P < 0.01) between the size ratio of broad-leaved forests and their corresponding productivity.

#### Correlation analysis of environmental factors with productivity, stand density, and structure diversity.

**Fig 2:**
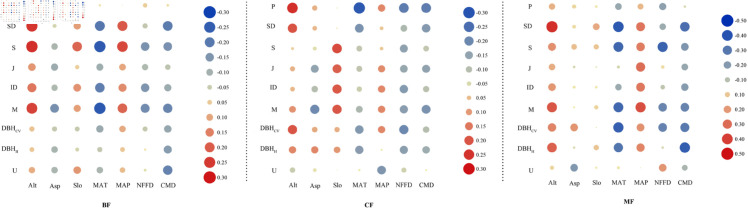
The correlation between environmental factors and productivity, stand density, and tree structural diversity. CF: Coniferous forest; BF: Broad-leaved forest; MF: Coniferous and broad-leaved mixed forest; P: Productivity; SD: stand density; Alt: Altitude; Asp: Aspect; Slo: Slope.

Topographical and meteorological factors of coniferous forests significantly affected productivity. Altitude was significantly positively correlated with productivity; whereas, MAT, MAP, and CMD were significantly negatively correlated (Fig 2). There was a no-table positive correlation between altitude and stand density for all three stand types; whereas, MAT and CMD were significantly negatively correlated with stand density. In coniferous forests, slope and NFFD were significantly correlated with composition diversity. Slope demonstrated a significant positive association with compositional diversity; whereas, NFFD exhibited the opposite trend. Meteorological factors showed a significant negative correlation between NFFD and diversity in size differentiation. In broad-leaved forests, altitude, slope, and MAP were significantly positively correlated with compositional diversity; whereas, slope orientation, MAT, and NFFD were significantly negatively correlated with CMD. Altitude and MAP exhibited a noteworthy positive correlation with compositional diversity in coniferous and broad-leaved mixed forests. However, MAT and NFFD were significantly and negatively correlated with CMD.

#### Analysis of factors affecting productivity in natural broad-leaved forest stands.

**Fig 3:**
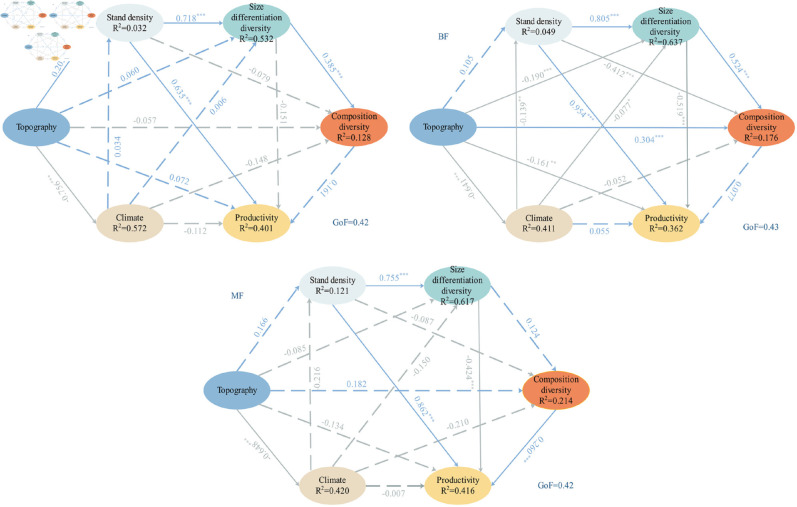
Path analysis results of stand productivity and its influencing factors. CF: Coniferous forest;BF: Broad-leaved forest;MF: Coniferous and broad-leaved mixed forest.

In coniferous forests, stand density and terrain were the main factors affecting productivity (Fig 3). Stand density significantly directly affected productivity ($r_{\partial }$= 0.635, P < 0.001) and terrain significantly indirectly affected productivity through stand density ($r_{\partial }$= 0.202, P < 0.05). However, stand structure diversity had no effect on productivity (size differentiation diversity: $r_{\partial }$ = −0.151, P > 0.05; composition diversity: $r_{\partial }$ = 0.161, P > 0.05).

In broad-leaved forests, terrain and size differentiation diversity were significantly negatively correlated with productivity (terrain: $r_{\partial }$ = −0.161, P < 0.01; size differentiation diversity: $r_{\partial }$ = −0.519, P < 0.001) and stand density was significantly positively correlated with productivity ($r_{\partial }$ = 0.954, P < 0.001). The effect of terrain on productivity was regulated by climate and size differentiation diversity. Terrain strongly negatively affected climate and size differentiation diversity (climate: $r_{\partial }$ = −0.641, P < 0.001; size differentiation diversity: $r_{\partial }$ = −0.190, P < 0.001). Climate significantly indirectly affected productivity through stand density and size differentiation diversity (stand density: $r_{\partial }$ = −0.139, P < 0.01; size differentiation diversity: $r_{\partial }$ = −0.077, P < 0.05).

In coniferous and broad-leaved mixed forests, there were significant correlations be-tween productivity and stand density ($r_{\partial }$= 0.862, P < 0.001), size differentiation diversity ($r_{\partial }$ = −0.424, P < 0.001), and composition diversity ($r_{\partial }$ = 0.260, P < 0.001). Stand density and composition diversity consistently exhibited positive correlations with productivity; whereas, size differentiation diversity showed a negative correlation. Furthermore, the effect of stand density on productivity was influenced by size differentiation diversity, as stand density also strongly positively affected size differentiation diversity ($r_{\partial }$ = 0.755, P < 0.001)).

#### Nonlinear test of the density-productivity relationship.

**Fig 4:**
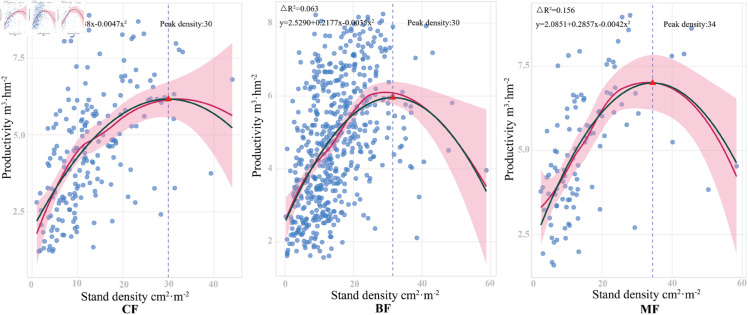
Testing the Nonlinear Relationship between Stand Density and Productivity. Pink area: LOESS smoothing curve (95% confidence interval); green solid line: quadratic polynomial fit.

Quadratic regression analysis revealed that the quadratic term coefficients for all forest types were negative, suggesting a unimodal relationship (Fig 4). The theoretical peak densities were as follows: BF $32\;{\text{cm}}^{2}\cdot {\text{m}}^{-2}$, MF $34\;{\text{cm}}^{2}\cdot {\text{m}}^{-2}$, and CF $30\;{\text{cm}}^{2}\cdot {\text{m}}^{-2}$, all of which fell within the observed density ranges (BF: 0–59, MF: 3–58, CF: 1–44). However, the improvement in explanatory power ($\Delta {\text{R}}^{2}$) of the quadratic model relative to the linear model was limited, with values of 0.063 for BF, 0.156 for MF, and 0.048 for CF. The LOESS non-parametric smooth curve further indicated that, within the observed density range, the density-productivity relationship exhibited an approximately linear trend, with only a slight curvature near the peak density. These findings suggest that, although the nonlinear relationship is statistically significant, its influence is relatively weak within the current data range.

## Discussion

During the research period from 2014â€’2019, productivity of coniferous and broad-leaved mixed forests in Zhejiang Province was significantly higher (21.35%) than that of broad-leaved forests; however, the increase in stand density was only 2.56% compared to that of broad-leaved forests. Coniferous and broadleaved mixed forests represent a multistoried stand structure, enabling trees to effectively utilize vertical spatial resources and reduce competition among tree species within the same layer [42]. Moreover, because of the distinct photosynthetic characteristics of coniferous and broad-leaved tree species, coniferous and broad-leaved mixed stands can more effectively exploit light resources [43]. Consequently, coniferous and broad-leaved mixed forests exhibited higher productivity than single-species broad-leaved or coniferous forests. CV serves as a crucial indicator for assessing trait dispersion levels, with larger CV values indicating greater dispersion across different forest stand types [44]. Regarding forest structure diversity, both broad-leaved forests and coniferous and broad-leaved mixed forests demonstrated significantly higher compositional diversity than coniferous stands. Overall, the diversity indicators for each component fell into medium or high variability categories. The CV for DBH among different forest stands was classified as a moderate variability indicator, and the DBH Shannonâ€’Wiener index and neighborhood comparison were considered weak variability indicators. Previous research has indicated that stand composition diversity and size differentiation are not solely affected by stand type but are also significantly affected by factors such as stand density, terrain conditions, meteorological patterns, and other environmental factors [45].

Based on a general linear model, there was an overall positive correlation between stand composition diversity and size differentiation in the three forest types and productivity. Among them, the impact of richness on productivity is much greater than that of other variables, highlighting the dominant influence of species richness on productivity. This phenomenon aligns with the niche complementarity effect [45–51]. Specifically, differences among tree species in phenology, root distribution, and photosynthetic characteristics allow for more efficient resource partitioning in both time and space, ultimately enhancing overall resource utilization efficiency. Local scale studies have different results based on specific factors such as region, forest type, and age [52,53]. It also further suggests that the relationship between species diversity and productivity may be limited by other factors such as species composition, stand development stage, and environmental factors [54,55]. The Pielou index of broad-leaved forests has a significant negative correlation with productivity.It has been shown that in broad-leaved forests, a few dominant tree species, leveraging their strong competitive ability for resource acquisition, play a key role in shaping community productivity [56]. However, this phenomenon is not observed in coniferous forests or mixed coniferous and broad-leaved forests. This may be attributed to the slow growth rates of coniferous trees, their conservative competition strategies, and the complex interspecific interactions in mixed forests, which collectively highlight the specific influence of tree species traits on productivity regulation. Similarly, studies by Padilla-Martínez *et al*. [57] and Hordijk *et al*. [58] also believe that as evenness increases, the positive impact of species richness on productivity will decrease. The coefficient of DBH variation and DBH Shannonâ€’Wiener index of the three forest stand types were significantly positively correlated with productivity.The complex diameter structure facilitates niche differentiation among trees. Small-diameter trees adapt to low-light conditions beneath the canopy and grow slowly, whereas large-diameter trees dominate the upper space and exhibit rapid growth. This size-based differentiation in growth strategies minimizes intraspecific competition, optimizes community resource partitioning, and consequently enhances overall productivity.

Changes in terrain and meteorology determine multiple aspects such as species distribution, composition, and growth rate. Species in natural communities have evolved over a long time period, forming a specific community structure in which species depend on and interact with their environment [59]. The correlation analysis showed significant correlation between the productivity of coniferous forests and environmental variables such as altitude, annual average temperature, frost-free period, and CMD. This suggests that its growth is jointly constrained by water balance and temperature conditions. In contrast, broad-leaved forests and mixed broad-leaved and coniferous forests show no significant correlation with topography and meteorological factors, likely due to the high adaptability of broad-leaved tree species to environmental changes [17,60]

Structure diversity responds to environmental heterogeneity and drives ecosystem productivity [61]. Considering the effects of regional environmental factors and stand density, the relationship between the structure diversity of coniferous forests and productivity was significantly weakened.This view is also supported by other similar studies [61–63], this suggests that the significant impact of terrain and stand density on productivity overshadows the role of structural diversity. Terrain factors indirectly affect the diversity and productivity of forest stand structures, leading to a non-causal correlation between stand structure diversity and forest productivity [26,61]. Many previous studies have believed that meteorology is an important factor affecting productivity [64], but in this study, climate only has a significant indirect effect on broad leaf forest productivity, indicating that the influence of climate change on forest productivity is mediated by forest type. Among the three forest stand types, density exhibits a significantly positive effect on productivity, which is consistent with the findings of Xiao *et al*. [65] and Brunner *et al*. [66]. Within a certain density threshold, mixed forests demonstrate a pronounced “over-production effect" driven by enhanced resource complementarity among tree species. Notably, in broadleaf forests and broadleaf-coniferous mixed forests, size differentiation diversity exhibits a significant negative influence on productivity. However, a significant positive correlation exists between size and species diversity, implying that the differentiated distribution of individual tree sizes may serve as a critical mechanism for maintaining community species diversity [67–69]. This also offers a novel perspective on the stability and development of forest ecosystems.

Although the quadratic regression analysis revealed a significant nonlinear (unimodal) feature in the density-productivity relationship, the explanatory power of the quadratic model was only marginally higher than that of the linear model ($\Delta {\text{R}}^{2}\;<\;0.16$). The LOESS smoothing curve indicated that within the observed density range, the density-productivity relationship generally followed an approximately linear trend. Notably, no pronounced curvature was observed near the theoretical peak density, suggesting that the linear relationship could adequately capture the association within the current study density range. Regarding model selection, assuming a linear relationship between density and productivity in the PLS-PM path model represents a reasonable simplification based on the characteristics of the research data. First, the approximately linear trend exhibited by the data provides empirical support for this assumption; second, the PLS-PM method demonstrates robustness against slight nonlinearities, ensuring the reliability of the model results to some extent. Nevertheless, given that the quadratic regression analysis has already highlighted the presence of nonlinear features, and the density gradient in this study did not encompass the highly competitive areas above $60\;{\text{cm}}^{2}\cdot {\text{m}}^{-2}$, future research should expand the density gradient sampling range, particularly focusing on high-density regions, and employ more sophisticated methods such as nonlinear structural equation modeling to further investigate the complex mechanisms underlying density-dependent responses. This would enhance theoretical understanding of the relationship between stand density and productivity and provide more precise guidance for forest management.

A better understanding of the structural diversity-productivity relationship at environmental scales can help identify co-benefits between promoting structural diversity and improving productivity, which has important implications for forest management and conservation practices [70]. Our research shows that the driving mechanism of natural mixed forests is restricted by stand types, and diversified forestry management measures should be adopted according to stand types to improve natural forest quality. For example, in coniferous and broad-leaved mixed forests, the effects of environmental factors on productivity may be weak.Artificially planting native tree species or implementing ecological thinning to optimize diameter structure and reduce size differentiation diversity can promote inter-specific niche complementarity, thereby enhancing stand productivity. For broad-leaved forests, moderately reducing size diversity (e.g., through selective removal of large-diameter individuals) can mitigate intraspecific competition and strengthen the resource acquisition capacity of dominant tree species, thus improving stand quality. Under climate change scenarios, such management strategies contribute to maintaining the ecological functions and long-term stability of broad-leaved forests. Overall, this study provides new information on the impact of forest stand structural diversity on forest productivity, the underlying mechanisms, and its interactions with environmental factors. However, this does not mean that our conclusion is indisputable and universal, because we have not yet combined factors such as soil and forest composition with productivity in our analysis. Moreover, the sample plot area of this study is $800\;{\text{m}}^{2}$, which may limit the expression of forest community composition and structure to a certain extent [71]. Therefore, we propose that future research should consider expanding the scale of sample plots, establishing additional long-term monitoring plots, and integrating multidimensional data, including soil, plant, and climate variables, to develop a more universally applicable model for predicting forest productivity.

## Conclusion

    There was a significant correlation between stand structure diversity and stand productivity in natural mixed forests in Zhejiang Province. The higher the species richness and the greater the complexity of DBH, the higher the stand productivity. There were notable variations in the relationship between environmental factors (topography and meteorology), stand productivity, and stand structure diversity across different forest types. A comprehensive analysis encompassing environmental factors, stand density, and stand structure diversity revealed that stand density exerted a direct effect on productivity in all three stand types. Terrain indirectly affected coniferous forest productivity through its effect on stand density, and directly affected broad-leaved forest productivity. Climate significantly indirectly affected broad-leaved forest productivity through stand density and size differentiation diversity. Regarding structure diversity, size differentiation had a pronounced negative effect on broad-leaved and coniferous and broad-leaved mixed forests; whereas, compositional diversity only exhibited a strong positive effect on coniferous and broad-leaved mixed forests. In summary, the relationship between stand structure diversity and productivity was regulated by environmental factors and stand density, with variations among different forest types. This study provides novel insights into the mechanisms driving forest productivity, which can inform policymakers when formulating rational forest management strategies to achieve sustainable utilization of forest resources while maintaining ecosystem stability.
